# On Laterally Perturbed Human Stance: Experiment, Model, and Control

**DOI:** 10.1155/2018/4767624

**Published:** 2018-05-02

**Authors:** Dan Suissa, Michael Günther, Amir Shapiro, Itshak Melzer, Syn Schmitt

**Affiliations:** ^1^Biomechanics and Biorobotics, Universität Stuttgart, 70569 Stuttgart, Germany; ^2^Stuttgart Centre for Simulation Sciences (SC SimTech), University of Stuttgart, Pfaffenwaldring 7a, 70569 Stuttgart, Germany; ^3^Institut für Sportwissenschaft, Friedrich Schiller Universität, Seidelstraße, 07749 Jena, Germany; ^4^Department of Mechanical Engineering, Faculty of Engineering, Ben-Gurion University of the Negev, Beer-Sheva, Israel; ^5^Schwartz Movement Analysis & Rehabilitation Laboratory, Department of Physical Therapy, Faculty of Health Sciences, Ben-Gurion University of the Negev, Beer-Sheva, Israel

## Abstract

Understanding human balance is a key issue in many research areas. One goal is to suggest analytical models for the human balance. Specifically, we are interested in the stability of a subject when a lateral perturbation is being applied. Therefore, we conducted an experiment, laterally perturbing five subjects on a mobile platform. We observed that the recorded motion is divided into two parts. The legs act together as a first, the head-arms-trunk segment as a second rigid body with pelvis, and the ankle as hinge joints. Hence, we suggest using a planar double-inverted pendulum model for the analysis. We try to reproduce the human reaction utilizing torque control, applied at the ankle and pelvis. The fitting was realized by least square and nonlinear unconstrained optimization on training sets. Our model is not only able to fit to the human reaction, but also to predict it on test sets. We were able to extract and review key features of balance, like torque coupling and delays as outcomes of the aforementioned optimization process. Furthermore, the delays are well within the ranges typically for such compensatory motions, composed of reflex and higher level motor control.

## 1. Introduction

How the human motor apparatus finds solutions to stabilize upright stance in the face of the body designed as a highly unstable multilinked-inverted pendulum has attracted the curiosity of modern scientists since at least 150 years [1], with quantitative analysis being intensified about 100 years ago by Murray et al. [2]. General neuromuscular control concepts have been derived therefrom by Asatryan and Feldman [3]; Feldman [4–6].

Until today, analyses of human upright stance have been mainly performed in the sagittal plane. There has always been a separation in analyses of quiet and disturbed human stance. Contrary to quiet stance, disturbed stance has earlier been identified to be at least a double-inverted pendulum (DIP) problem by Nashner and McCollum [7]; Horak and Nashner [8]; Yang et al. [9]; Kuo [10]; Kuo et al. [11]; Runge et al. [12]; Kuo [13]; Fujisawa et al. [14]; Kiemel et al. [15]; Roth et al. [16] in the sagittal plane. Since about ten years ago, however, most researchers bore in mind a single-inverted pendulum (SIP) model by Geursen et al. [17] as an accompanying paradigm Winter et al. [18]; Morasso and Schieppati [19]; Morasso and Sanguineti [20]; Gage et al. [21] for their examinations of quiet stance in the sagittal plane. When examining quiet stance, even DIP models by Geursen et al. [17] equipped with just one additional degree of freedom (DOF) were not given attention until about a decade ago. Then, modern findings by Creath et al. [22]; Hsu et al. [23]; Saffer et al. [24]; Pinter et al. [25]; Günther et al. [26–28]; Yamamoto et al. [29] had finally pinpointed that quiet stance is in fact a multijoint phenomenon for which it takes at least triple-inverted pendulum (TIP) models to gain an understanding of its dynamics in the sagittal plane by Günther et al. [30]; Günther and Wagner [31]. To the best of our knowledge, only one study by Günther et al. [32] has examined rotations around the long axis of the body so far. And few studies by Bonnet et al. [33, 34] focused on the lateral (or frontal, resp.) plane, for which Day et al. [35] provided an analytic benchmark.

It can be expected that stance dynamics in the lateral plane, be it externally disturbed or undisturbed (quiet), should show some similarity to those in the sagittal plane, with the primary difference that stance width is clearly higher than foot length, and the knees are rather compliant in sagittal but stiff in lateral direction. This leads us to the assumption that the DIP would be a well-suited model for lateral stance, accepting that the TIP is a well-suited “minimal model” for the sagittal plane, and that the knee joint is “missing” in the lateral direction. Anyone, gently pushed aside at the shoulder, or as in our perturbations case, undergoing a lateral shift of the ground, would probably respond with just a compensatory bending movement in the lower spine and hip, maybe combined with some load shift between both legs. The similarity to disturbed stance in the sagittal plane would be a DIP-like movement plus a shift in center of pressure (COP). Thus, aiming at an enhanced understanding of control strategies for lateral disturbances, we developed the aforementioned DIP model for simulating responses to such disturbances. We did this purposefully without modeling physiological structures for neural signal excitation and force generation (muscle-tendon units).

Instead, we restricted ourselves to formulating abstract joint torque generators, as simple as possible. In particular, we based our control for both joints on an approach that can be seen as a “classical engineering method”: proportional-differential (PD) controllers. This abstract and simple approach allows a line of reasoning and inferences just reversal to an a priori implementation of physiologically based signal-processing and force-generating: initially neglecting all physiological properties (of control) but allowing some of them as a possible result of an optimization process can help to reveal their significance for the motor task under consideration. Accordingly, in this study, we chose the outlined approach to try and identify principal strategies and feedback loop properties supportive for stabilizing laterally disturbed human stance. The chosen approach particularly allows to disclose interdependences of control parameters like beneficial ratios (compare, e.g., Yang et al. [9]; Alexandrov et al. [36, 37]) and their relation to mechanical body design or beneficial time relations between sensor signals, actuator inputs, and actuator outputs. Based thereon, conclusions are possible relating to basic properties that may be imprinted into physiological and anatomical structures due to fundamental requirements for realizing the motor task (i.e., to the structures' function): the mechanical body design (e.g., mechanical coupling through biarticular muscles) and the physical constraints (e.g., reaction times) the body is exposed to during the intended motor task.

## 2. Material and Methods

### 2.1. Inverse Kinematic Model

**Figure 1:**
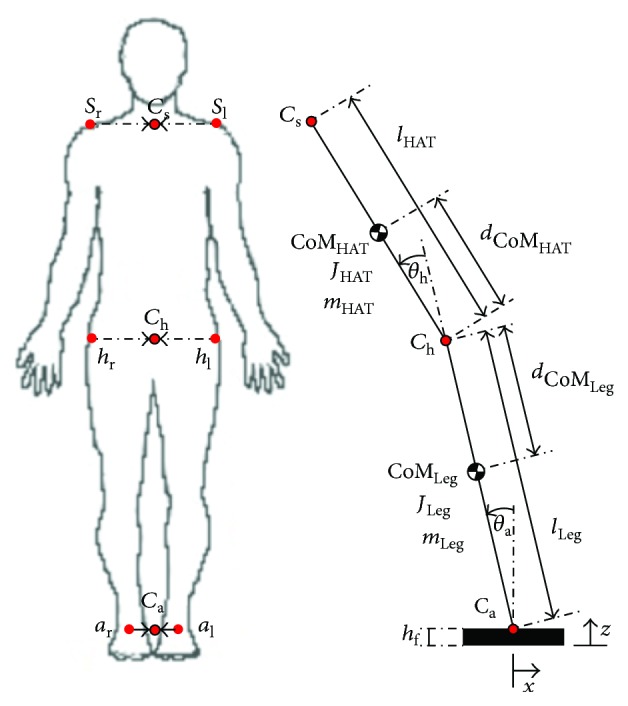
The marker positions (as in the experiment) at ankle, hip, and shoulder and their corresponding centers and the DIP model with: feet (height *h*_f_), ankle joint (defined by *C*_a_ and angle: Θ_a_), Leg segment between *C*_a_ and *C*_h_ (defined by: *l*_Leg_, COM_Leg_ which is at *d*_COM_HAT__ from *C*_h_ and by *m*_Leg_, and inertia *J*_Leg_), hip joint (*C*_h_, Θ_h_), and the HAT segment between *C*_h_ and *C*_s_ (*l*_HAT_, COM_HAT_ at *d*_COM_HAT__ from *C*_h_, and by *m*_HAT_, *J*_HAT_).

As can be seen in Figure 1, the planar mechanical model consists of three rigid bodies. The feet are represented by a box ((0.2 × 0.2 × 0.02)[m], 1 kg). They are to be considered, a graphical component of the model, aside from lifting the ankle joint to a height of *h*_f_ = 0.02 m. Furthermore, the DIP is composed of two rigid bodies: “Leg,” lumping both legs into one and “HAT,” depicting the head, arms, and trunk of the human body. The segments are defined between the centers (*C*_a_, *C*_h_, and *C*_s_) by their masses, lengths, center of mass (COM) positions and inertia. Part of the anthropometric data were measured (i.e., total body height, segment lengths, and total body mass), while the segment masses and COM positions were calculated according to Winter [38]. Moments of inertia (2D) of the model segments with respect to their centers' of mass are given in the following equation (infinitely thin, but rigid rod):

(1) $$\begin{array}{c} J_{Leg}=m_{Leg}\cdot l_{Leg}^{2}\cdot \frac{1}{12}\cdot I,\\ J_{HAT}=m_{HAT}\cdot l_{HAT}^{2}\cdot \frac{1}{12}\cdot I, \end{array}$$

where *I* is the identity matrix. The COMs are located at distance 0.45 · *l*_Leg_ (leg) and 0.5 · *l*_HAT_ (HAT) from the hip joint point, respectively (Figure 1).

The kinematics of the model are as follows: the “HAT” segment rotates around the upper end of the “Leg” segment (*C*_h_) and the “Leg” segment around the center of the feet (*C*_a_). The feet are laterally translated from their initial position (=perturbation). Thus, the mechanical model's three DOFs are the two angles Θ_a_ and Θ_h_ and the translational DOF *x* of the foot. Note that we did not constrained the DOFs which occur in the human body, for example, by ligaments. We assumed that Θ_a_ and Θ_h_, as well as their first two derivatives and the acceleration of the perturbation $\ddot{p}$ (acting on *C*_a_) may be sensed. These system states (and derivatives) are combined to form the input to the controllers that generate the torques applied to the ankle and hip. For further reading on the construction of the mechanical model utilizing the experimental data (i.e., marker positions), the physical properties of the model, and the implementation in SimMechanics, we refer to Appendix B.

### 2.2. Control

The DIP is perturbed by a motion signal (${\left[p,\dot{p},\ddot{p}\right]}^{T}$) along *x*, deviating the feet laterally from their initial position. For our simulations, the perturbation signal *p* is experimentally obtained (lateral translation of *C*_a_) and filtered by a PT2 low-pass filter at 6 Hz (as all experimentally obtained data). Note that the filter also provides the two derivatives $\dot{p},\ddot{p}$ (for filtering, see Appendix A.4).

As an abstraction of muscle force acting in humans ankle (A) and hip (H), joints are controlled by joint torques (*M*_A_, *M*_H_). Different control approaches are applied in order to represent central nervous system (CNS) strategies (note that I and IIa/b are reference strategies):

(I) There is no control, simulating “no reaction” of the CNS to the perturbation (i.e., free falling/collapse of the body in response to the perturbation).
(II) A “P(200)D(20)” controller with set point 0°, for both joints: (a) without delay and (b) with 20 ms delay. This “technical” controller is representing the intuitive “control to 0°” strategy, similar to a SIP control strategy. Note that delays are introduced due to finite signal propagation velocities in the nervous system.
(III) A dynamic spring-damper system is assumed by (2), based on that human posture is controlled by muscle-tendon complexes and sensor feedback, including proprioception and information about the perturbation:
  (2) $$\begin{array}{c} M_{a}^{ds}\overset{{delay}_{1}}{\leftarrow }M_{1}=-k_{1}\cdot y_{1}-d_{1}\cdot {\dot{y}}_{1}+q_{1}\cdot \ddot{p},\\ M_{h}^{ds}\overset{{delay}_{2}}{\leftarrow }M_{2}=-k_{2}\cdot y_{2}-d_{2}\cdot {\dot{y}}_{2}+q_{2}\cdot \ddot{p}. \end{array}$$

The parameters *X* = [delay_1/2_, *k*_1/2_, *d*_1/2_, *q*_1/2_] are acquired by unconstrained nonlinear optimization (see Appendix A.5), minimizing the squared error between given angular trajectories (i.e., experimental data) and those obtained in the simulation of the model (*E*_ang_):

(3) $$\begin{array}{c} \underset{X}{\min}\;E_{ang}=\underset{X}{\min}\;\sum_{i}\left({\left(\Theta_{A,i}^{\exp}-\Theta_{A,i}^{sim}\right)}^{2}+{\left(\Theta_{H,i}^{\exp}-\Theta_{H,i}^{sim}\right)}^{2}\right), \end{array}$$

where *i* is the sampling points. Following approaches for *y*_1/2_ for the controller III (2) are implemented to compare (a) uncoupled torques, (b) torques coupled by coupling angles one-to-one, and (c) torques coupled by coupling angles with the mixing ratios *β*:

(4a) $$\begin{array}{c} \left(IIIa\right)\text{: }y_{1}=\Theta_{a},y_{2}=\Theta_{h}; \end{array}$$

(4b) $$\begin{array}{c} \left(IIIb\right)\text{: }y_{1}=y_{2}=\Theta_{a}+\Theta_{h}; \end{array}$$

(4c) $$\begin{array}{c} \left(IIIc\right)\text{: }y_{1}=\beta_{11}\cdot \Theta_{a}+\beta_{12}\cdot \Theta_{h},y_{2}=\beta_{21}\cdot \Theta_{a}+\beta_{22}\cdot \Theta_{h}. \end{array}$$

In approach (IIIc), the weight factors (i.e., mixing ratios) *β* equal the inertia tensor of the mechanical model (fitted to the current trials participant, not to be confused with the inertia of the model segments *J*_Leg/HAT_), numerically calculated by the inverse dynamics approach, which is explained in the next Section 2.2.1.

#### 2.2.1. Weights by Inverse Dynamics

Performing an inverse dynamics approach, the measured angular trajectories (Θ_a_^exp^(*t*), Θ_h_^exp^(*t*)) were considered moving set points, of high gain (hg) PD controllers (*P* = 2000, *D* = 20, and *N* = 100), to be retraced. The resulting torques *M*_a_^hg^ and *M*_h_^hg^ are the bases for the computation of the weight factors.

Alternatively starting from the Newtonian perspective, the joint torques may be simply approximated by a dependency of angular accelerations with *M* = *I*∗*α* where *I* is a moment of inertia and *α* an angular acceleration. With this ansatz

(5) $$\begin{array}{c} M_{a}^{LSQ}=\beta_{11}\cdot {\ddot{\Theta }}_{a}+\beta_{12}\cdot {\ddot{\Theta }}_{h}+\beta_{31}\cdot {\ddot{p}}_{sim},\\ M_{h}^{LSQ}=\beta_{21}\cdot {\ddot{\Theta }}_{a}+\beta_{22}\cdot {\ddot{\Theta }}_{h}+\beta_{32}\cdot {\ddot{p}}_{sim}, \end{array}$$

we assume an inertia tensor *β* of dimension [3 × 2]. The upper [2 × 2] part of *β* (given in kg · m^2^), corresponding to the contributions from the angular accelerations in the ankle and hip joints, approximates the 2D inertia tensor of the DIP (parametrized according to the participant). It is used as weighting factor between the ankle and hip angle in the states *y*_1/2_ of the controller (IIIc), see (4c). The lower part including *β*_31_ and *β*_32_ (given in kg · m) is not used further and thus ignored in the following.

*β* is found from minimizing the squared and summed error of both Newtonian torque approximations *E*_tot_ = *E*_a_^2^ + *E*_h_^2^, over all time steps ts (LSQ):

(6) $$\begin{array}{c} E_{a}=\sum_{ts}\left|M_{a}^{hg}-M_{a}^{LSQ}\right|,\\ E_{h}=\sum_{ts}\left|M_{h}^{hg}-M_{h}^{LSQ}\right|. \end{array}$$

In (4c) (IIIc), *β*_11,12,21,22_ (kg · m^2^) are multiplied with Θ_*i*_ (deg); thus, *y*_*i*_ has the unit (kg · m^2^ · deg). Since in (2) the left hand side has to be a torque (Nm = kg · m^2^/s^2^), the unit for *k*_*i*_ is given by (1/deg · s^2^), for *d*_*i*_ is given by (1/deg · s), and for *q*_*i*_ is given by (kg · m), since *q*_*i*_ is multiplied with an acceleration.

### 2.3. Experiment

Experiments were conducted to (i) calculate the parameters of our assumed controllers, (ii) check the robustness of these controllers, and (iii) verify validity of the model. Five university students, four (1, 3–5) healthy and one (2) with occasionally light knee, hip, and back pain (i.e., rather “stiff” back and hip joint), of very similar physique participated in the experiment: all male, 26 ± 1.5 yrs, 69.4 ± 5.03 kg, 1.756 ± 0.03 m.

The risk to fall for the healthy young adults is minimal, when perturbing stance with the BaMPer system; thus, no additional safety measures had to be taken. All participants gave informed consent. The experiments took place, all on the same date, in the “Schwartz Movement Analysis and Rehabilitation Laboratory,” Faculty of Health Sciences, Ben-Gurion University of the Negev, Beer-Sheva, Israel. The subjects took part on a purely voluntary basis. Studies on the BaMPer system were approved by the Helsinki Committee of Barzilai University Medical Center, Ashkelon, Israel (ClinicalTrials.gov registration number NCT01439451).

The perturbations were applied using the BalanceTutor© by MediTouch Ltd. which is based on the BaMPer system developed by Shapiro and Melzer [39]. Motion analysis was provided by the Ariel Performance Analysis Software (APAS version 10.100.0.1) running on a lab PC, utilizing two camcorders (Cannon NTC ZR100) with a frame rate of 59.94 fps. Six reflective LED markers (1 cm diameter) were placed on the participants' front: one on each foot, placed directly above the ankle joint, two at the hip, on the most protruding points of each sides of pelvic bone, and another two at the shoulder, placed at the most protruding point of the clavicle. Additional markers were placed on the moving part of the treadmill in order to capture the perturbation, as well as a global reference marker on the lab wall.

The participants were individually led into the lab and instructed to stand barefoot, upright, in hip-wide stance (on individually placed ground markers) on the BalanceTutor. They were asked to fold their hands behind their backs and keep them and the feet at their initial positions, throughout the whole experiment, if possible. Participants knew that they would receive lateral perturbations at their base of support, repeatedly during a period of a couple of minutes, executed through a shift of the laterally movable treadmill, to either the left or right.

The set of at least 12 perturbations was of increasing magnitude (each to the left and right, for 10/14/18 cm distance with acceleration capped to either 150 cm/s^2^ or 200cm/s^2^) and was executed with no prior notice. Thus, perturbations were random in direction and time of onset, to mimic unexpected loss and recovery of balance. If arm movement or stepping was recorded, the trial was repeated once.

### 2.4. Average Controller and Prediction of Trials

From the set of valid experimental trials, we built a training (*S*_tr_) and two test sets (*S*_te_, *S*_te_^(4)^). Training is done by optimizing the controller, individually for each trial and by then building the average over all obtained parameters. The final controller is then verified by predicting on the test sets. Due to strong qualitative similarities, we did not distinguish between the different perturbation magnitudes and directions, as well as the (in magnitude) different responses of the participants.

### 2.5. Overview: Data/Workflow

**Figure 2:**
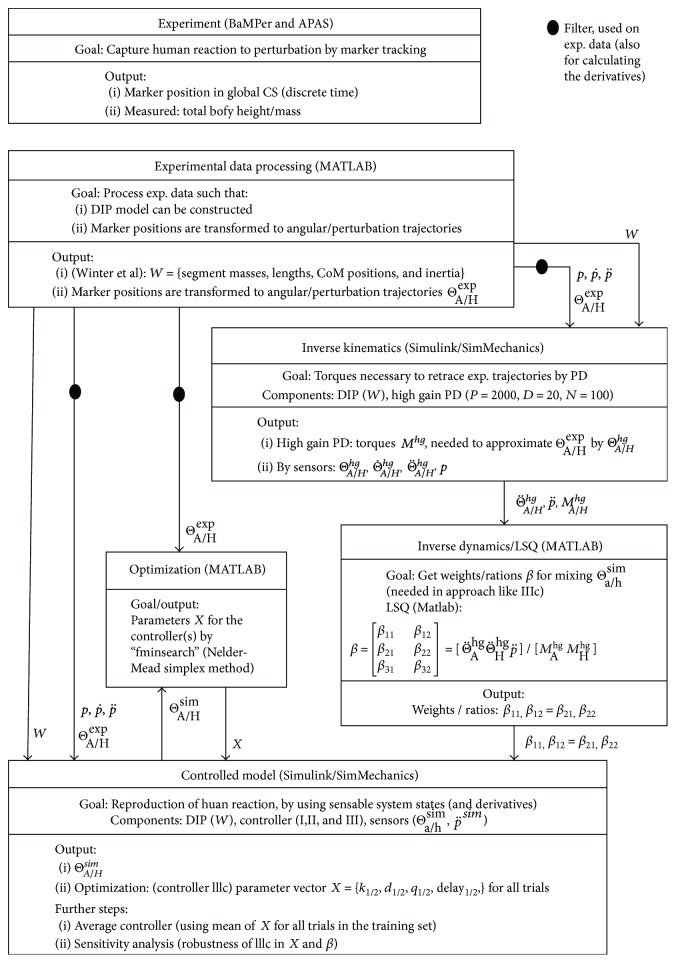
Data and workflow of the project. This sketch illustrates what was done, in which order and with the help of which tools. Large subscript A/H are used if the trajectory is meant, and small subscript a/h are used if only one value (e.g., sensor signals at a certain point in time).

Figure 2 depicts the data and workflow of the whole project.

## 3. Results

### 3.1. Experiment

#### 3.1.1. Perturbation

**Figure 3:**
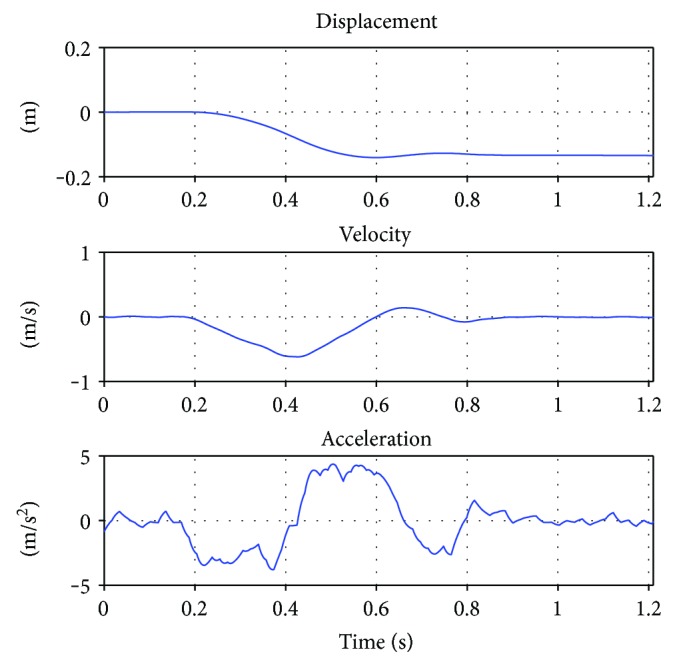
Filtered perturbation signal. For each trial, a qualitatively similar signal, containing change in position, velocity, and acceleration, was used. Quantitatively the perturbation signals differed in direction, thus to the left or right and in displacement and velocity.

**Figure 4:**
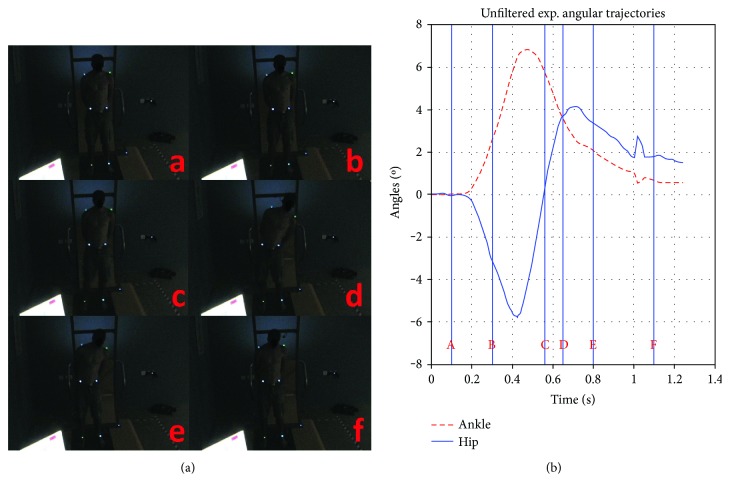
(a) Typical frames of the experiment, from a single trial, at the distinguished phases (A, B, C, D, E, F). (b) Corresponding trajectories of the hip and ankle angle, horizontal lines correspond to the phases. See also Appendix D for further reading.

A set of 12 perturbations was executed in the experiment. They differ in magnitude and direction but not qualitatively. In Figure 3, the motion signal used for perturbing the DIP model is shown for a test case. The test case is a trial by participant (5) (which is 14 cm displacement and 200cm/sec^2^ acceleration trial; from now on referred to as (5)14200lt), as this is closest to the average in the experiment. Trial (5)14200lt is shown in Figure 4 as an example. The whole motion, from preperturbation (A), to perturbation (B, C), to initial participant response (D), to full balance recovery (E, F), is explained in the following.

#### 3.1.2. Results of the Experiment and Strategy-Dependent Variations

A simple segment length criterion (see Appendix A.2) identified two participants (1, 2), which deviated from the basic strategy, invalidating almost all their trials due to length changes of up to 15 cm in the Leg segment and up to 6 cm in the HAT segment. This was confirmed visually by the video, where in nearly all trials, participant (1) is extremely bending the knees and rotating the hip and spine horizontally, resulting in heavy A/P tilting. Participant (2) on the other hand acts extreme stiff around the lumbar spine and hip, resulting in a lift of one or both feet, see also Section 4.2.

Loss of balance (i.e., falling) indeed never occurred, as participants used arm raising or stepping if needed, resulting in 71/87 (81.6%) initially marked as “successful trials” (no arm movement and intentional stepping detected). Aside from that, within the participants (3, 4, 5) exhibiting a “proper” execution of the strategy (e.g., no A/P movement), only 5/36 trials (13.9%) had to be excluded.

**Figure 5:**
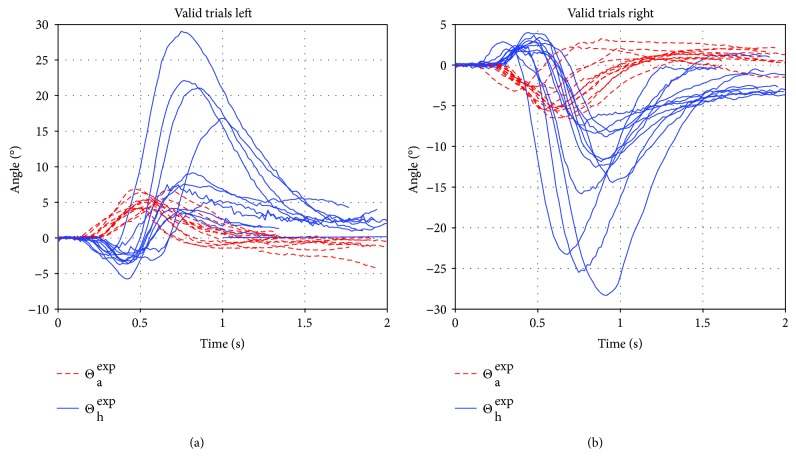
Unfiltered angular trajectories for valid trials of the experiment. In the (a) plot are the perturbations to the left, and in the (b) are perturbations to the right. The solid line (blue) is the hip the dashed line (red) is the ankle angle trajectory. All the “extreme” hip angle peaks stem from participant (4).

Participant (4), as can also be seen in Figure 5 exhibited bending of the lumbar spine (just like participant (1) and in contrast to (2, 3, 5)). However in his case, this led to changes of the segment length within the error margin *r*, and thus, his trials were not excluded. Here, the hip angle excursions were up to 3.5 times higher than those of participants (3 and 5).

Within the valid trials, the ankle bends to a peak 5–7.5° while the “extended hip” (i.e., hip + lower spine) bends to a peak of up to 10° (34° for participant (4)). The higher variety within the hip stems from bending of the lumbar spine, which was accepted if segment lengths did not change significantly and thus lumped into the “hip” DOF. However, bending of the spine in the coronal plane, together with bending of the hip in the sagittal plane (often together with a rotation in the transversal plane), occurs in some trials, in all participants and thus, leading to greater segment length changes. Such trials were excluded from our data set as our planar model could not cope with them. In Figure 5, valid trials of the experiment are plotted, while in Table 1, the mean of all max angles and angular velocities over all valid trials is given for each participant.

### 3.2. Model and Control

#### 3.2.1. Reference Controller (I) and (IIa/b)

**Figure 6:**
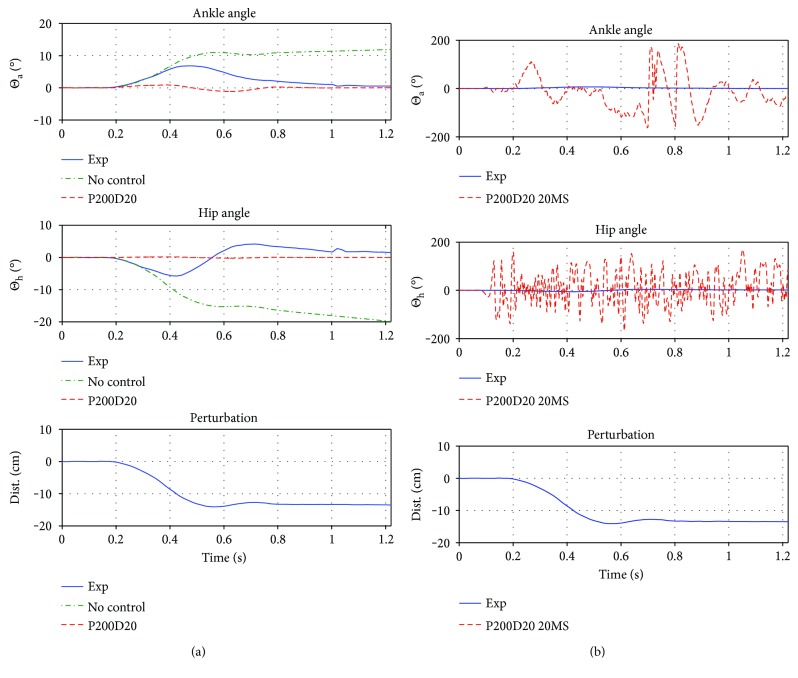
(a) Experiment trajectory in blue, “no control” (I) in red, and PD control (IIa) in dashed red. (b) Experiment trajectory in blue and PD control with 20 ms delay (IIb) in dashed red. First plot is the ankle trajectory, second is the hip, and third is the perturbation. While with no control applied, the system obviously collapses, also simple PD control, while being able to stabilize was not able to match the angular trajectories of the experiment. If a delay is introduced, the system becomes highly unstable. The PD controller used here is the MATLAB : Simulink build in PID controller with *P* = 200, *D* = 20, *I* = 0, and *N* = 100.

In Figure 6, we are presenting a comparison of results obtained by using “no control” (I), “P(200)D(20)” (IIa), and “P(200)D(20) + 20 ms delay” (IIb), to the results obtained in the experiment. While, obviously, none of the approaches can reproduce the experimental data, it is still possible to stabilize the system using a common PD controller (which is making the model behave very similar to a SIP), however, only if there is no delay introduced in the sensor signals or the controller output. Note that delay on either the output or delay on the sensor signals leads to a similar result (Figure 6(b)) and that delays are inevitable in humans. Consequently, any simple PD control must fail to stabilize in the biologically restricted system.

#### 3.2.2. Controller (IIIa/b)

**Figure 7:**
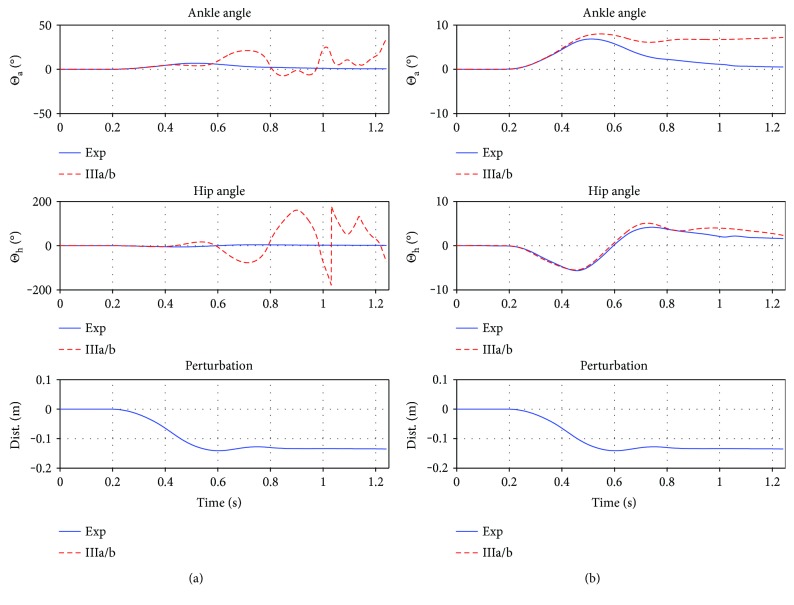
(a) Controller IIIa, best fit, with an remaining error of 45.73 deg/ts. (b) Controller IIIb, best fit, with an remaining error of 2.7 deg/ts. Both fitted to the same experimental trial (5)14200lt. First plot is the ankle trajectory, second is the hip, and third is the perturbation. While IIIa fails to stabilize (no torque coupling), even the introduction of simple one to one torque coupling (IIIb) is enough to (1) stabilize the system and (2) almost reproduce the experimental trajectories.

Figure 7 depicts the best fits of the first two approaches (IIIa, b) in comparison to the test case (5)14200lt. The parameters of the dynamic system (IIIa/b) are optimized such that Θ_a/h_^sim^(*t*) are fitted to Θ_a/h_^exp^(*t*) of a single experimental trial ((5)14200lt), which is done by unconstrained nonlinear optimization.

The approach (IIIa) was invalidated, as it completely fails to reproduce the angular trajectories (not stabilizing the mechanical model at all).

The approach (IIIb), which, by using a linear combination of both angles, is a lot closer to the sought solution, is nevertheless also invalid: the remaining error after optimization is big, and the controller is unable to stabilize the mechanical model.

#### 3.2.3. Controller (IIIc)

First, the weights for *y*_1/2_ (see (4c)) are calculated by an inverse dynamics approach via least square optimization. They are specific to the participant (not to the perturbation), that is, *β*^(5)^ is the same for all trials of participant (5), within small numerical differences. Thus, the weights can be calculated a priori, individually for each participant. The weights of participant (5) are *β*_11_ = 1.12, *β*_12_ = *β*_21_ = 0.23, *β*_22_ = 0.06 (kg · m^2^). Note that it is a result of the simulation that *β*_12_ = *β*_21_, within an accuracy 0.1%. The before mentioned unconstrained nonlinear optimization algorithm is used to find well-suited control parameters *X* = {*k*_1_, *d*_1_, *q*_1_, delay_1_, *k*_2_, *d*_2_, *q*_2_, delay_2_}.

“Optimal” (i.e., local minimum) values for ((5)14200lt) are

(7) $$\begin{array}{c} k_{1}=0.72\frac{1}{\deg\cdot s^{2}},\\ d_{1}=2.04\frac{1}{\deg\cdot s},\\ q_{1}=0.83\;kg\cdot m,\\ {delay}_{1}=0.06\;s,\\ k_{2}=0.66\frac{1}{\deg\cdot s^{2}},\\ d_{2}=1.7\frac{1}{\deg\cdot s},\\ q_{2}=0.57\;kg\cdot m,\\ {delay}_{2}=0.13\;s. \end{array}$$

**Figure 8:**
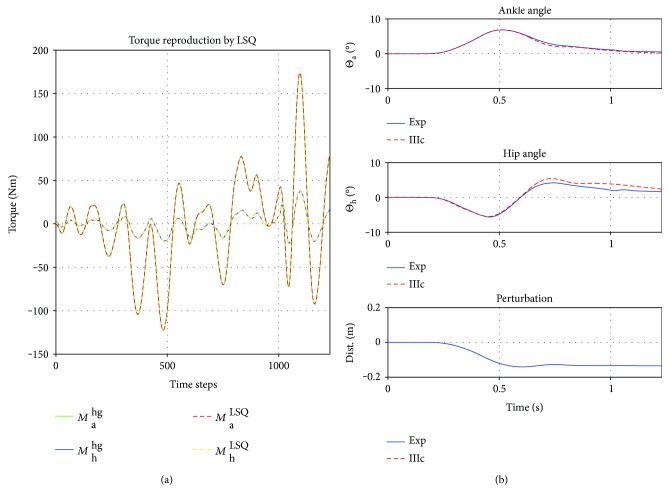
(a) The torques generated by the high gain PD: *M*_*i*_^hg^ versus *M*_*i*_^LSQ^, *i* ∈ {*a*, *h*} with virtually *M*^LSQ^ = *M*^sim^. *y*-axis is torque in Nm over time steps of the simulation on the *x*-axis. (b) Response of controller IIIc. The best fit, with an remaining error of 0.16 deg/ts. First plot is the ankle trajectory, second is the hip, and third is the perturbation. Finally, the controller IIIc with torque coupling according to the numerical inertia tensor *β* realized the almost exact reproduction of the experiments.

The left plot in Figure 8 depicts the “exact” reproduction of *M*_a_^hg^ and *M*_h_^hg^ by the LSQ algorithm (used to find *β*, remember (5)) and the trajectories of the corresponding controller, utilizing the upper [2 × 2] part of *β*, in comparison to the experiments on the right.

### 3.3. Average Controller “*IIIc*_*avrg*_”

Of the 31 valid trials, we took 10 for the training set *S*_tr_ and 11 for the test set *S*_te_. Participant (4) is not part of training and test set due to his great (quantitative) deviation in strategy (extreme hip angles by bending the lower spine a lot more than other participants); he was treated separately by checking the performance, by the set *S*_tr_ parametrized average controller, on all his trials.

In Table 2, the average of all parameters after optimization on the training set is listed. The column “Error” is the average error made by the controller optimized for each specific trial in *S*_tr_. This value is useful comparing the average controller error on the training and test set to the error made by the controller optimized to specific trials. The mean error thus is 0.4 deg/ts, while the best fit is trial (5)14200lt with 0.18 deg/ts and the worst fit is trial (3)14150lt with 0.58 deg/ts.

Note that the average is taken over the controller parameter set *X*, not over the *β* values. The latter are participant specific and thus have to be changed according to the current participant in every simulation (see also Robustness Analysis and Discussion).

When applying controller IIIc without the information about the disturbances (*p*_*i*_ = 0 kg · m, IIIc.1) and additionally even without the explicit angular information (*k*_*i*_ = 0(1/deg · s^2^), IIIc.2), qualitatively nothing changes on the training set except for an increase in error by  100%. This means that, in principle, only angular velocity information is needed for stabilization and qualitative correct reproduction of the trials, all other information just enhance the fit and prediction quality.

#### 3.3.1. Training

We applied the average controller (parameters given in Table 2), to the trials both in the training and the test set and evaluated the corresponding errors.

**Figure 9:**
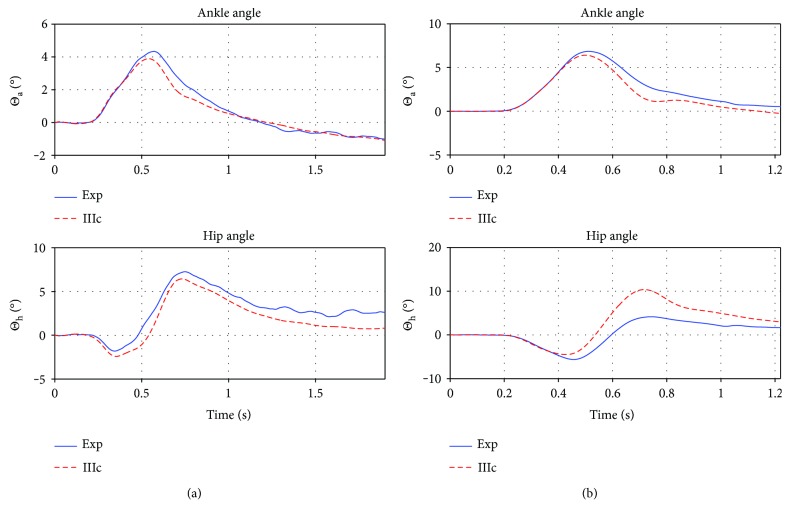
Best (a) and worst (b) prediction on *S*_tr_. Plotted are the ankle and hip angle in deg. over time in seconds. The red/dashed lines are the experimental trials, and the blue/solid lines are the corresponding simulation of controller IIIc_avrg_. On the test set, the averaged controller ranged in quality of reproduction between those two trials. Obviously the prediction is good, since the test set trials were used to find the parameters *X*.

The mean error of IIIc_avrg_ on *S*_tr_ was 1.35 deg/ts (assuming a mean trial length of 1.6 sec and thus 1600 timesteps (ts) per angle making a total of 3200 ts for the trial). The greatest error made was on the trial (5)14200lt with an error of 2.17 deg/ts. Trial (3)10150lt was the best, with 0.87 deg/ts. The respective fits are shown in Figure 9.

#### 3.3.2. Test

**Figure 10:**
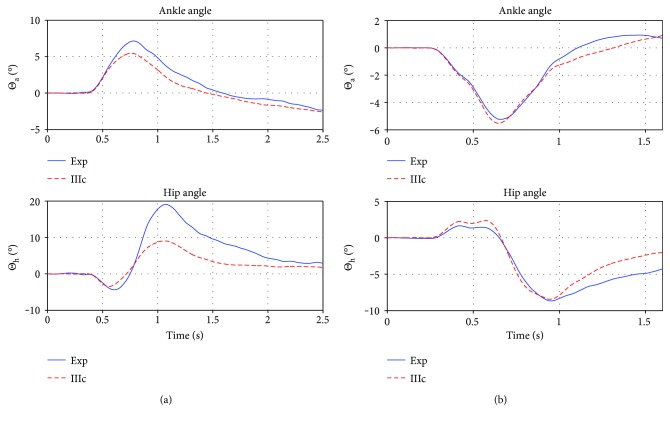
Best (a) and worst (b) prediction on *S*_te_. Plotted are the ankle and hip angle in deg. over time in seconds. The red/dashed lines are the experimental trials, and the blue/solid lines are the corresponding simulation of controller IIIc_avrg_. Both trials are qualitatively correct and the system is stabilized. The best prediction on the left shows a slight underestimation in the ankle and a slight overestimation in the hip at the end of trial, predicting the rest of the trajectory with high precision. The right (worst) prediction on the test set underestimates the experimental trajectory midtrial, while the beginning and end are predicted fine.

The average controller was tested on *S*_te_, the mean error on the test set was 2.24 deg/ts (again mean trial length = 1.6 sec), while trial (5)14150rt was the best fit with an error of 0.89 deg/ts, and the second best was (5)10150lt with 1.52 deg/ts and the greatest error was made on trial (3)18200lt with an error of 3.32 deg/ts, while the second greatest was 2.43 deg/ts in (3)18150lt. The average error without the worst two and the best two fits was 1.7 deg/ts. The best and worst fit are again shown in Figure 10.

#### 3.3.3. Participant (4)

**Figure 11:**
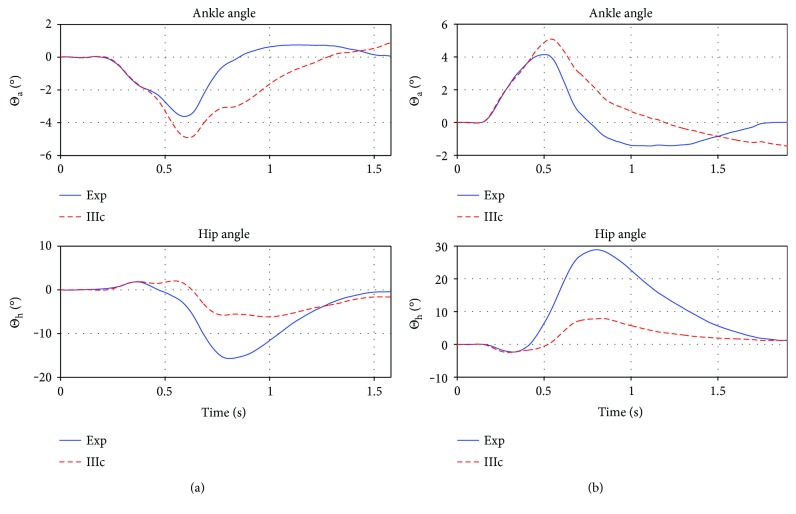
Best (a) and worst (b) prediction for participant (4). Plotted are the ankle and hip angle in deg. over time in seconds. The red/dashed lines are the experimental trials, and the blue/solid lines are the corresponding simulation of controller IIIc_avrg_. Without considering participant (4) in finding the control parameters *X*, the controller is able to predict his trials, however with a considerable amount of under and overestimation, but not to that point where qualitative change in behavior or instability is observed.

We furthermore compared the average controller's performance to all nine trials of participant (4) (*S*_te_^(4)^). While the time courses of the ankle angle fits are always reasonable, the great hip angle deflection cannot be reproduced by the controller fitted to the training set. The error in the least square step (calculating the ratios *β*) is already two magnitudes higher than with the other participants (usually between 20–200, for participant (4); however, errors are in the thousands). Also the error when applying the average controller is 1-2 magnitudes higher than for the other participants (mean error is 6.12 deg/ts, best fit is for trial (4)14150rt with 3.24 deg/ts, and worst fit is for (4)14150lt with 7.55 deg/ts). In comparison, the controller optimized to the specific trials of participant (4) has an mean error of 0.77 deg/ts, with the best fit for (4)14150rt with an error of 0.3 deg/ts and the worst fit for trial (4)18150lt with 1.14 deg/ts. Nevertheless, the average controller manages to reproduce the experiment trajectories qualitatively, which is shown in Figure 11, and the model restabilizes in response to the disturbance.

### 3.4. Robustness Analysis

Finally, we conducted a robustness analysis, by varying controller parameters of the controller IIIc optimized to a single trial (again (5)14200lt) manually, in order to assess the robustness of the simulated responses with respect to the parameters of the controller. Robustness in our context is defined as still showing qualitatively the same time course of the responses and stability (low remaining error between simulation and experiment at the end of balance recovery, *t* = *t*_end_).

#### 3.4.1. Parameters *X*

The controller IIIc has proven to be robust, not at last since it can cope with a range of perturbation strengths and varying kinematics in responses. Variations in the parameters {*k*_1_, *k*_2_, *q*_1_, *q*_2_, delay_1_, delay_2_} of *X* more than 10% did not change qualitative behavior of the controller and still allowed for a good fit to the experimental curves to be made, even in the extreme case, where more than one parameter is varied by more than 10%. If only one parameter from {*k*_1_, *k*_2_, *q*_1_, *q*_2_, delay_1_, delay_2_} is varied by 10%, the error goes up to about 0.55 deg/ts. By varying all parameters in *X* at once by ±10% (thus not changing the ratio between *d*_1_ and *d*_2_) the error grows from 0.18 deg/ts to 0.86 deg/ts, no loss of stability and no significant change in kinematics occur. The ratio between *d*_1_ and *d*_2_, that is, the ratio between the velocity contributions to both joints is the most critical one and should be varied by at most 5%; thus, if the ratio between the coupled torque generation of the controllers (which is mainly dependent on *d*_1_, *d*_2_) is changed, qualitative change and loss of stability is the consequence, for example, variation of *d*_1_ by 5% (for a fixed *d*_2_) leads to an error of 1.16 deg/ts, and a variation of 10% leads to qualitative change in behavior (especially in the hip) with an error of 2.4 deg/ts. For all trials, with a small enough remaining error (local minimum with an error ≤0.75deg/ts after optimization to a specific trial), the ratio was found to be the same, namely *d*_1_/*d*_2_ = 1.14 ± 0.03. Thus, also if one varies all parameters of the ankle controller {*k*_1_, *d*_1_, *q*_1_, delay_1_} by 10% without changing the hip controller parameters, the error goes to 2.62 deg/ts with qualitative change in response.

Furthermore, in unconstrained nonlinear optimization, several (equivalent) local optima may be found

(8) $$\begin{array}{c} X^{*}=\arg\underset{X}{\min}\;E_{ang}=\underset{X}{\min}\;\sum_{i}\left({\left(\Theta_{A,i}^{\exp}-\Theta_{A,i}^{sim}\right)}^{2}+{\left(\Theta_{H,i}^{\exp}-\Theta_{H,i}^{sim}\right)}^{2}\right), \end{array}$$

where *i* is the sampling points. So for example, optimization for (5)14200lt returned the set of parameters *X*_1_ = (0.72(1/deg · s^2^), 2.04(1/deg · s), 0.83 kg · m, 0.06 s, 0.66(1/deg · s^2^), 1.7(1/deg · s), 0.57 kg · m, 0.13 s) with a remaining error of 0.16 deg/ts and the second local minimum *X*_2_ = (1.40(1/deg · s^2^), 1.79(1/deg · s), 0.62 kg · m, 0.06 s,1.23(1/deg · s^2^), 1.57(1/deg · s), 0.59 kg · m, 0.15 s) with a remaining error of 0.18 deg/ts, depending on initial conditions *X*_0_. However, note the ratio *d*_1_/*d*_2_ = 1.12 for *X*_1_ and 1.14 for *X*_2_ (please refer to Figure 8 to see the controller with *X*_1_), which is within the margin for both optimization results.

As can be seen in Table 2, the delays of the ankle were 60 ms in average (represented by delay_1_ of (2)) and the delays in the hip 171 ms in average (represented by delay_2_ of (2)). Even if the controller seems pretty robust in the delays (mentionable is that they were pretty robust if decreased but hit a limit very fast when increased at around +15%), all delays were found closely to these values in the optimization, with an at least twice as high delay in the hip as in the ankle. The balance compensatory responses in the hip are “reflex-like” responses. They are long latency responses that involved the central nervous system (CNS) and not only the spinal cord, as compared to the delays at the ankle joint.

#### 3.4.2. Ratios *β*

However, regarding the angular mixing ratios *β* (remember *β* is dependent on the participants' individual physique, for example, inertia and geometry, see Table 3), the controller shows altered qualitative behavior with loss of stability if varied too much, and even for small variations, a remaining angular deviation from zero (or from the experimental curve) remains at the end of simulation (*t* = *t*_end_) which is too great. This was again a result firstly obtained from the construction of the average controller IIIc_avrg_, where in a first approach also the mean values of all *β* values in *S*_tr_ were used, while the DIP model still adapted segment data according to the current trial. This practice was leading to qualitatively wrong (and unstable) controller behavior (not fitting well to the experiment trajectory).

Furthermore, we manually tried to determine robust ranges for the parameters *β* or more precise for the ratios *β*_11_/*β*_12_ and *β*_22_/*β*_21_ and thus the ratios between mixing angles for the coupled torques, by varying the parameters *β*_12_ = *β*_21_. For the test trial (5)14200lt, *β*_12_ = *β*_21_ cannot exceed variations of ±2.5%, without undergoing qualitative changes (e.g., only positive/negative angles) and for variations greater than ±5% loss of stability. Analogous outcome for variation of *β*_11_ or *β*_22_. This is reflected in the errors for varying *β*_11_ or *β*_22_: for the original parameters, it is 0.18 deg/ts and for a variation of 2.5% it is 0.68 deg/ts, exhibiting slight under and over fitting of the angular trajectories. For a variation of 5%, it is 1.18 deg/ts with a remaining error at the end of balancing, and for a variation of 10%, it is 2.5 deg/ts, with loss of stability. If all values in *β* are changed by the same ±10%, the error grows to 0.87 deg/ts. Thus, the controller still works, if the ratios of angular mixing are left untouched (which are thus mainly depended on *β* and *d*_1/2_ in *X*).

Remember, the error values alone are not sufficient to detect qualitative change. Only by looking at the trajectories one may determine if qualitative change has indeed occurred. The error is merely an indicator, which works good to determine if a fit or prediction was correct (low error values) but not if it was incorrect just because the error values are high(er).

## 4. Discussion

### 4.1. Generality of the Controller

The controller IIIc is not only used to reproduce trials (to which it was parametrized to), but also to predict trials not used in training (remember training error of 1.35 deg/ts). The quality of prediction for this test set is absolutely within the range of the fitting to the training set (even if the numerical error value on the test set with 2.24 deg/ts or 1.7 deg/ts is higher than in training, the fits are very good as can be seen, comparing Figures 9 and 10).

Notable is that the training consisted of trials from two participants, which were used regardless of their magnitude, over the full range of perturbations tested in the experiment, also ignoring the direction. As this was not only possible, but also leading to good results, the strong correlation between trials of different magnitudes and participants could be verified: the same control scheme, with one parameter set could be used. The full range of reactions to the perturbation, tolerable by the controller, is also illustrated by Figure 5. We are certain that for a greater number of trials, participants, and magnitudes, this mean result would converge to a smaller error than possible now, under the prior mentioned premises, and to a certainly much more accurate prediction if only one magnitude of perturbations is used with many participants. It would also be possible to extend the found control law such that it considers the perturbation strength to a higher degree and apply different magnitudes of reaction accordingly.

However, we have chosen this advance to prove some generality of the controller (e.g., robustness over a broad range of trials). Even for participant (4), where a variation of strategy could be observed (much greater angles in the hip due to extended spinal bending in l1-l5), the average controller (IIIc_avrg_) was able to qualitatively reproduce the behavior. If participant (4), however, would have been part of training, the controller would fit much better to these trials, but also worse for the other two participants which did not bend the lower spine to that extend.

The extreme numerical values of the errors on the set of participant (4), *S*_te_^(4)^, show, if again comparing Figures 9, 10, and 11, that the error value is/can be used only as a proof of correct response and only as an indicator for possibly incorrect response.

### 4.2. Limits of This Study

First of all, we have to state that an uncertainty quantification (UQ) was not performed in a mathematical rigorous manner.

The present study contains several potential sources of errors which could lead to wrong conclusions. Most importantly, the choice of an appropriate model is crucial to analyse the measured data. Moreover, model parameters have to be found and are subject to an uncertain quantification. Further, the experimental data itself is also known to be a typical source of errors.

Some sources of errors are easy to mention, for example, the data recording had an error in the marker tracking of approximately 1 cm, which lead to maximum errors in the calculated segment lengths of up to 2 cm. Please refer to Appendix A.2 (invalid trials and exceeder of the “basic” strategy) for a further discussion.

Other potential biases are not so trivial to judge. In particular, the level of detail of the model is a crucial first-hand choice. For this study, we chose a double-inverted pendulum (DIP) model. However, simpler single-inverted pendulum (SIP) or more complex models are also used in literature for representing human upright stance balance Günther et al. [30]; Günther and Wagner [31]. Among the more complex models, triple-inverted pendulum (TIP) models are used for lateral stance control, but it exist also models incorporating even a higher complexity, like multiple multibody systems, taking the spinal movement into account, for example, Rupp et al. [40].

For the case of simpler models, like the SIP model, they can be eliminated due to two reasons: First, the experimental results of our study show that all participants exhibited roughly the same magnitude of movement in the hip and ankle joint following the perturbation. A SIP would depict only one of these degrees of freedom, totally ignoring the dynamics in the other. Second, the simulated controller IIa (simple PD control) is not reproducing the experimental results and also fails to stabilize, if small delays are introduced (IIb, Figure 6). This controller behaves roughly like a SIP with almost all movement occurring in the ankle joint.

More interestingly from our perspective would be the case of using more complex models for the analysis. First, currently, some trials had to be excluded from the analysis due to the length criterion (see Appendix A.2) based on anterior/posterior movement. Using a 3D model including 3D joints, the variation of the balance strategy (extension of the basic planar strategy) could have been pictured. By that, the biological system benefits by (i) increasing robustness by further lowering the COM. Furthermore, by using a 3D TIP, the hip and ankle would also move in the A/P direction and the knee joint could be included for even greater increase of robustness too. (ii) The biological system further benefits by a higher flexibility, which is equivalent to a situational dependent change of the balance strategy. Second, all participants exhibited movement in (at least) the lower spine. Some subjects were very stiff in the lower spine region. In some, however, one could see with the naked eye that the lower spine bending contributed more to lateral angular excursions of the trunk than rotations around the hip joints. A model considering a flexible upper segment, in total or in part, would improve the reproduction and depiction of this motion.

The last source of uncertainty we want to mention is the commonly known fact that unconstrained nonlinear optimization may end up in several local minima that may or may not be roughly equivalent (see also Results). So better solutions cannot be fully excluded.

### 4.3. Restabilization Strategy

In the present work, we were able to establish and review key factors of balance. Therefore, we want to especially discuss the need for coupling of torques and the role of delays imposed on balance control. We were also able to draw a line of reasoning between our findings and the eigenmovement theory [37], which is discussed together with the predicted delays at the second part of this section.

**Figure 12:**
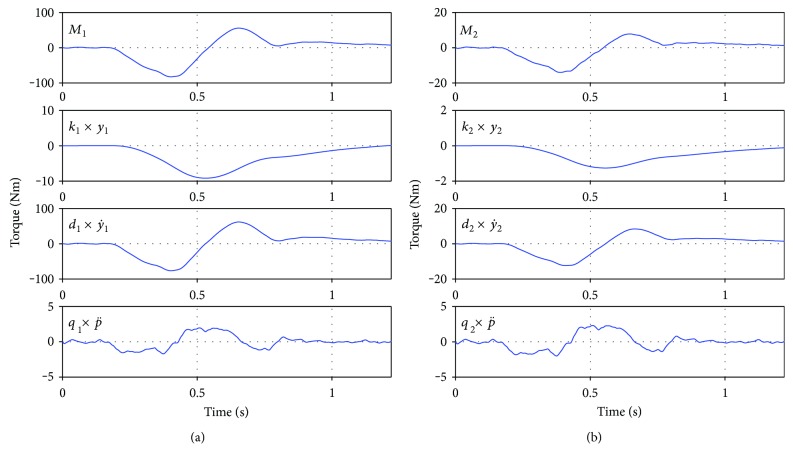
Controller IIIc_avrg_ in the test trial ((5)14200lt). (a) *M*_1_ (undelayed *M*_A_) full and split into components. (b) *M*_2_ (undelayed *M*_H_) full and split into components. As can be easily seen, the primary influence on the applied torques always stems from the velocity contributions, while the other two contributions (displacement and perturbation acceleration) just slightly alter the final torques.

The necessity for torque coupling, where one joint torque needs to take into account the other joint torques in order to account for their changes and influence on the other joints, was proposed and reviewed by earlier studies [9, 37] and was again confirmed by our work. We realize this by sensor signal coupling, utilizing *β* in (4c) and thus in the generation of motor actions (2). We found that in the latter (2), the most critical parameters are the damping parameters *d*_1_ and *d*_2_ as they influence torque generation the most ($\dot{y}$ has the highest influence on the torques, see Figure 12). Especially the ratio between *d*_1_ and *d*_2_ was found to be roughly *d*_1_/*d*_2_ = 1.14 ± 0.03 within our trials and to ensure a certain ratio between the two torques in the ankle and the hip, which was preestablished by the ratios *β*, variations of one of the *d* parameters while fixing the other should not exceed 5%. Aside from that critical relation, the possibility of variation of the parameters *X* is intuitive since a slight change of reaction strength and time (i.e., onset) should not be a problem. If they would, stability in an uncertain system like the upright standing human body would not be possible. Thus, the observed robustness in *X* is no surprise. Also, the smaller robustness in *β*, which contains information about the inertia and geometry (which are rather fixed properties of the mechanobiological system), is clear. Already slight change in these ratios results in qualitative changes and instability. Thus, with *β* and the relation *d*_1_/*d*_2_, which are together defining the coupling of torques, being the least robust, the necessity of (correct) coupling and relations between torques of different joints becomes obvious. The simulation yielded *β*_12_ = *β*_21_, which states that into each joint torque, the same amount of information about the other joint (angle and angular velocity) is taken into account.

For our motor task, the found parameters of *X* and *β* further impose that *y* is mainly generated by utilizing ankle angle information (*y*_1_: *β*_11_ is roughly 5 times larger than *β*_12_ and *y*_2_: *β*_21_ is roughly 4 times larger than *β*_22_) and that the torque in the ankle is 5 times larger than in the hip as well (again the ratio between *β*_11_ and *β*_21_). Numerical values of the fixed relation between torques in different joints were given for A/P balance in [9], with ratios for hip to knee at 1.5 and ankle to knee at 2. Further, it was stated that biarticulated muscles “have the architectural capacity to elicit fixed proportions of two torques about the joints they cross.” According to [9], the delay between ankle and hip could be the hip “waiting” for the ankle reaction to start, in order to react correctly.

The predicted delays between kinematic signals and joint torques for controller IIIc (about 60 ms for ankle and 170 ms for hip) are intriguing, because 60 ms in particular seems to be a good reflexion of a neuromuscular delay between changes in muscle proprioceptive signals (muscle spindles, Golgi tendon organs) and corresponding changes in motor output, that is, muscle force or joint torque, respectively.

Propagation velocities in afferent and efferent, mammalian motor nerves are about 100 m/s (typ I) or 50 m/s (typ II) ([41]). The distance between muscle and motoneuron is roughly 0.5 m for human leg muscles and between muscle and brain about 1.5 m. The fastest conceivable way of delayed sensor information feedback from mechanical actuator or joint sensors back to the actuator in a biological system is via the monosynaptic pathway muscle-motoneuron-muscle. According to the above-mentioned distances, the total monosynaptic delay would thus be about 10 ms (and 30 ms in the brain case). Another synaptic delay is added at the neuromuscular junction (between the axon's end terminal and muscle surface: about 1 ms Katz and Miledi [42]). A third contribution to neuromuscular delay seems to be the prevailing one in humans: the electromechanical delay (EMD) as the time passing between the onset of a finite change in electrical muscle surface stimulus beyond the junction, measurable, for example, by a surface electromyography (EMG), to the onset of a measurable, finite change in muscle force ([43]). EMD was found in human's quadriceps muscle to be 50 ms ([44]), when starting from inactivity. Signal processing in neural nets like the motoneuron pool in the spinal cord or in the cerebellum introduces additional contributions to feedback loop delays.

Monosynaptic delay in leg muscles via the motoneuron pool is thus about 60 ms and matches well the delay between joint angle proprioception and ankle torque as found here to be effective for restabilization. This fact is an indication of the neuromuscular design of human beings to be well adapted to the mechanical requirements of regulating human upright posture which is a salient feature in the animal world due to the unique mechanical design of the human foot. A hip joint torque contribution effective for the same requirements is, however, even more delayed by further 110 ms. Are there indications that may help to understand this further delay?

A delay between distinct subfeatures of motor commands was found to be effective in a different, yet related, motor task: voluntary trunk bending of humans during upright standing ([37, 45]). More precisely, Alexandrov et al. [37] had found for voluntary trunk bending that (i) a superposition of A- and H-eigenmovement is optimal for fulfilling the bending movement and stability at once and that (ii) a delay between the onset of both eigenmovements is essential (A-eigenmovement must start earlier than H-eigenmovement). A- and H-eigenmovements are two motor commands based on differently weighted combinations in joint angular shifts (the eigenvectors of the mechanical, three-link system). In our case, the task is different, somehow an inverse problem: the body has to be restabilized after a disturbance, whereas in the experiments of Alexandrov et al. [45], humans bent their trunks voluntarily. In our case, obviously a delay between the joint torque contributions *within* a near-A-eigenmovement (i.e., ankle and hip contributions) must become effective (difference between 170 ms and 60 ms).

In the first instance, this finding of a delay between local joint torque contributions when compensating a lateral disturbance is clearly different from time sequencing the onset of A- and H-eigenmovements. However, the approach of Alexandrov et al. [37] to motor synthesis based on utilizing the eigenvectors of the mechanical system may be appropriate as a general background with explanatory potential, if particularly combined with ideas ([46]) about the mutual relation of sensory signals and motor commands within the three-dimensional, redundant neuromusculoskeletal system and their potential processing in the central nervous system. Our findings seem to provide some further support for use of the eigenvector approach. Since, the restabilization strategy (2) with (4c) identified in our study is very similar to the A-eigenmovement identified by ((10) in [37]) in trunk bending (“A” refers to the ankle joint). To see this, we have to compare our joint torques *M*_a_^ds^ and *M*_h_^ds^, respectively, to the first and third components of their vector *u*_A_, that is, we first have to compare the ratios of our weightings (Table 3) of ankle and hip angles in (4c) *β*_11_/*β*_12_ ≈ 5 as well as *β*_21_/*β*_24_ ≈ 4—determining *M*_a_^ds^ and *M*_h_^ds^ in (2) by *k*_1_ · *y*_1_ and *k*_2_ · *y*_2_ (*k*_1_ ≈ *k*_2_ ≈ 0.7 s^2^/deg, see (7)), respectively—to the ratio *w*_A,1_/*w*_A,3_ ≈ 3 of the first and third components in the A-eigenvector *ω*_A_ in ((10) in [37]). Second, due to *k*_1_ ≈ *k*_2_, a ratio *M*_a_^ds^/*M*_h_^ds^ ≈ *β*_11_/*β*_21_ ≈ 5 of the angle-dependent joint torque contributions in *M*_a_^ds^ and *M*_h_^ds^ for the same angle deflections in ankle and hip compares to the ratio of first and third components *u*_A,1_/*u*_A,3_ ≈ 4.7 in the left equation (11) in [37]. Altogether, we may thus call our identified strategy a “near”-A-eigenmovement because the ratios *β*_11_/*β*_12_ and *β*_21_/*β*_22_ of the ankle and hip angle-dependent contributions to the joint torques *M*_a_^ds^ and *M*_h_^ds^, respectively, in this strategy are slightly distorted as compared to *w*_A,1_/*w*_A,3_ in the A-eigenmovement (see left equation (10) in [37]), whereas eventually regarding the ratio of the angle-dependent ankle and hip torque contributions themselves (compare *β*_11_/*β*_21_ and *β*_12_/*β*_22_ to *u*_A,1_/*u*_A,3_ in the left equation (11) in [37], there is almost no difference to the A-eigenmovement in terms of the joint torque ratio *M*_a_^ds^/*M*_h_^ds^.

Velocity-dependent contributions are an implicit part of the eigenvector-based approach in Alexandrov et al. [37]. They are explicitly reflected in the restabilization strategy (2) with (4c) for compensating lateral disturbances as treated here. The ratio of linearly velocity-dependent contributions to *M*_a_^ds^ and *M*_h_^ds^ is approximately *d*_1_/*d*_2_ · *β*_11_/*β*_21_ ≈ 5.5 times higher for *M*_a_^ds^ than for *M*_h_^ds^, which thus makes little difference to the ratio of angle-dependent contributions because *d*_1_/*d*_2_ ≈ 1. With angular velocities that are typically about the same in the hip and the ankle (see Table 1 with maximum angular velocities), the ratio of velocity-dependent contributions to *M*_a_^ds^ and *M*_h_^ds^ is also similar to the ratio of the angle-dependent parts. Remarkably, the velocity-dependent torque contributions (i) dominate the whole restabilization strategy (2) with (4c) by far (compare magnitudes of angle- and velocity-dependent contributions in Figure 12) and (ii) the strategy works even without any “knowledge” of the control system about the disturbance (*q*_*i*_ = 0) and the angular position (*k*_*i*_ = 0). Using the word “work” implies that this strategy restabilized the model in the simulations of all trials available, including even those by participant (4) exhibiting exceptional angular amplitudes, albeit the intermittent and final states deviating from corresponding measured ones more than when using angular information (*k*_*i*_ ≠ 0). All this indicates that sensor information about rates of mechanical state variables is of fundamental importance for human upright stance. Positional information takes a back seat as does explicit disturbance sensation.

As the velocity-dependent torque contributions are time-delayed, they do not represent mechanical friction or “damping” but are rather forecasts (see McMahon [47], p.154–156) of an angle-dependent torque contribution that corresponds to a first-order prediction of a change in joint angle ${\Delta \Theta }_{j}=d_{i}/k_{i}\cdot {\dot{\Theta }}_{j}$ (*j* = *a*, *h*) predicted to occur for a given angular velocity ${\dot{\Theta }}_{j}$ that will, however, not take mechanical effect before an instant delay_*i*_*s* later. Such velocity-dependent torque contributions compensate for potential deflections in the future that would be much larger than will actually occur as long as their compensatory effect is in fact stabilizing. Here, these torque contributions take mechanical effect delayed by times comparable to the typical time to reach the maximum angular deflections (100 … 200 ms). Introducing motor actions like (2) which are not due to locally instantaneous mechanical effects but based on delayed sensor signals of mechanical variables fed into an information transmission channel to be decoded and transformed into a proportional actuator force later on is information-costly ([48]) but enables access to a greatly enlarged diversity of movement solutions.

A rather simple control system based on the system's mechanical eigenvectors and one or another tailored time delay can be evidently set up for initiating voluntary movements or compensating significant disturbances of an *N*-link-inverted pendulum without escalating the movement system into resonance disaster. It should be checked whether a recently suggested stabilization strategy for a TIP model simulating quiet human stance in the sagittal plane ([31]) is in fact similar, related, or even more or less identical to the eigenvector approach. It seems an obvious thing to take further steps and broaden the check for how general this idea of motor control based on mechanical eigenvectors is. Since, to the best of our knowledge and somehow surprisingly, this approach has solely been explicitly applied to human stance [9, 10, 13, 49–51] in the field of model-based, biomechanical movement synthesis of multilink systems so far. We cannot see obvious reasons, however, why cyclic or goal-directed movement should not be a promising field alike for eigenvector-based investigations to their motor control.

## Figures and Tables

**Table 1:** Mean and standard deviation of the maximal absolute angle and angular velocity for the ankle and hip for all valid trials.

Participant	Θ_a_^max^	Θ_h_^max^	${\dot{\Theta }}_{a}^{\max}$	${\dot{\Theta }}_{h}^{\max}$
(3)	5.26 ± 1.94 deg	10.38 ± 5.42 deg	19.88 ± 7.09deg/s	60.29 ± 26.98deg/s
(4)	3.73 ± 1.55 deg	22.40 ± 8.76 deg	24.00 ± 8.73deg/s	106.7 ± 39.45deg/s
(5)	5.62 ± 0.9 deg	7.45 ± 2.57 deg	25.94 ± 4.08deg/s	50.14 ± 10.42deg/s

**Table 2:** Average parameter set *X* for controller IIIc on the training set *S*_tr_ referred to by IIIc_avrg_.

Controller	Error	*k*_1_	*d*_1_	*q*_1_	delay_1_	*k*_2_	*d*_2_	*q*_2_	delay_2_
IIIc	0.35 deg/s	1.18 (1/deg · s^2^)	1.97 (1/deg · s^2^)	0.45 kg · m	0.06 s	0.85 (1/deg · s^2^)	1.73 (1/deg · s)	0.53 kg · m	0.17 s
IIIc.1	0.54 deg/ts	1.27 (1/deg · s^2^)	2.53 (1/deg · s)	—	0.06 s	1.19 (1/deg · s^2^)	2.06 (1/deg · s)	—	0.12 s
IIIc.2	0.55 deg/ts	—	2.53 (1/deg · s)	—	0.06 s	—	2.05 (1/deg · s)	—	0.12 s

**Table 3:** Participant specific ratios *β*.

Participant	*β*_11_	*β*_12_ = *β*_21_	*β*_22_	*β*_11_/*β*_12_	*β*_22_/*β*_21_
(3)	1.34 kg · m^2^	0.25 kg · m^2^	0.06 kg · m^2^	5.45	0.24
(4)	1.03 kg · m^2^	0.20 kg · m^2^	0.05 kg · m^2^	5.06	0.26
(5)	1.12 kg · m^2^	0.23 kg · m^2^	0.06 kg · m^2^	4.8	0.27

## Appendix

### A. Technical Details

#### A.1. Discretization and Solver

Experimental trajectories are discretized in time according to the camera frame rate with 60 data points per second. All output trajectories in MATLAB : Simulink (e.g., measured controller inputs Θ_A/H_^sim^) are time wise discretized with the (fixed) solver step size of 0.001 s and thus 1000 data points per second. A fixed step size (0.001 sec) fourth-order Runge-Kutta (ode4) solver was used.

#### A.2. Invalid Trials and Exceeder of the “Basic” Strategy

The aforementioned negligence of the *y* component obviously leads to great segment length changes if a trial exhibits too much movement in the A/P plane, for example, through bending of the knee, hip, and spine. Also not noticed stepping resulted in segment length changes (primarily in the “Leg” segment), s.t. trials with exceeder of the studied behavior, which were not identified by sight, could be identified in this step.

Trials exhibiting these “unwanted” motions are detected, by using a cutoff error on segment length changes of |*r*_HAT_| = 2 cm (3.5% of segment length) and |*r*_Leg_| = 2.5 cm (5% of segment length).

Note that those values were chosen such that they ignored inaccurate marker tracking/noise and are also in accordance to the variety of initial segment length values within the set of trials of a single participant (i.e., inaccurate measurements, calibration, tracking are within the error margin). Such that only trials with segment length changes due to change in strategy (stepping, extreme spine, hip, or knee bending) are discarded.

#### A.3 Training and Test Sets

The training set contains the following trials *S*_tr_ = [(3)10150lt, (3)14150rt, (3)10200rt, (3)14150rt, (3)18150rt, (5)14150lt, (5)14200lt, (5)14200rt, (5)18150rt, (5)18200rt].

The test set contains *S*_te_ = [(3)14200lt, (3)14200rt,(3)18150lt, (3)18200lt, (3)1820rt, (5)10150lt, (5)10200lt, (5)18150lt, (5)10150rt, (5)10200rt, (5)14150rt].

Trials in the set for participant (4) are *S*_te_^(4)^ = [(4)10150lt, (4)10200lt, (4)14150lt, (4)14200lt, (4)18150lt, (4)10150rt, (4)10200rt, (4)14150rt, (4)18150rt].

#### A.4 Filter

The filter used is a PT2 element (low-pass state variable filter[REF]) which was realized in Simulink according to the following transfer function

(A.1) $$\begin{array}{c} G_{f}\left(s\right)=\frac{\omega^{2}}{s^{2}+2\cdot \xi \cdot \omega \cdot s+\omega^{2}}, \end{array}$$

with the symbols representing signal s, filter frequency *f* = *ω*/2 · *π*, signal damping term *D* = 2 · *ξ* · *ω*, and filter coefficient *K* = *ω*^2^.

Used values are filter frequency *f* = 6 *Hz* and damping coefficient *ξ* = 0.707. The filter also offers to setup an initial condition, which is *x*_0_ = 0 *m* in our work.

This filter provided a good method to get corresponding (filtered) derivatives to a filtered signal, without having a time delay between the signal and its derivatives. This did also provide the SimMechanics actuator with the needed perturbation signal $\left[p,\dot{p},\ddot{p}\right]$ and the controller with the derivative of *y*.

However, there exists a delay between the “input” (i.e., the signal to be filtered, e.g., “perturbation position” *p*, *θ*_a_^exp^, *θ*_h_^exp^) and the filtered “value” (i.e., the filtered signal), such that, signals that have to be compared have to be filtered and thus delayed by the same magnitude (the delay on all signals, for ((5)14200lt), is 38 ms at 6 Hz).

#### A.5 Optimization

We use a MATLAB built-in unconstrained nonlinear optimization algorithm (fminsearch, based on Nelder-Mead simplex). However, the absolute value of the delays is used in the controller to hinder negative delay (impossible), which is done by an “abs()” block in Simulink, and thus not influencing optimization. Since nonlinear optimization can get stuck in local minima, choosing a good set of initial parameters *X*_0_ is a bit tricky, and various solutions may exist.

### B. Building the Model

#### B.1 Segment Mass, COM, and Inertia Properties

The model masses and COM locations are calculated according to Winter [38], and they are as follows.

HAT segment: considering the masses of head, neck, arms, and trunk has a weight of 0.678 in relation to total body mass (segment mass/total body mass). The length of the upper segment was considered as the length of the trunk only. The neck and head were not considered in the model segment lengths and therefore also not in COM positioning of the model so that the COM of the upper segment of the model is located at a distance of 0.5 times HAT length from the hip.

Leg segment: considering masses of both feet, shanks, and thighs has a mass ratio of 0.161 per leg, which is 0.322 times total body mass, for the lower segment of the model. The distance of the Leg segments COM from the hip is 0.447 times leg length.

#### B.2 Mechanical Model Generation from Marker Positions

The following depicts the creation of the model from the experimental data, and please also refer to Figure 1.

As a first step, the discrete raw marker positions are exported from APAS via Excel to MATLAB, the time step is 0.017 s (i.e., camcorder frame rate), and a usual trial is 1.5 ± 0.5 s long. Ignoring the A/P movement in this step, by neglecting the whole *y* component, results in 2D data of the motion within the M/L plane, to which the DIP is restricted. A marker position is thus composed of (*x*, *y* ≡ 0, *z*), where *x* is the (lateral) horizontal deviation from the origin and *z* is the vertical deviation from the origin. All positions are given in meters.

As can be seen in Figure 1, the three centers *C*_a_, *C*_h_, and *C*_s_ between the corresponding markers a_l/r_, h_l/r_, and s_l/r_ are simply calculated by

(B.1) $$\begin{array}{c} C_{i}=\frac{\left(i_{r}+i_{l}\right)}{2},\;i\in \left\{a,h,s\right\}. \end{array}$$

Then, segment lengths (*l*_Leg_, *l*_HAT_) are calculated as the vertical distance between the three centers (*C*_a_, *C*_h_, and *C*_s_), and the model constructed such that the initial position (in the global CS) of the ankle joint is at (0, 0, 0.02)[m], the hip is at (0, 0, 0.02 + *l*_Leg_)[m], and the shoulder is at (0, 0, 0.02 + *l*_Leg_ + *l*_HAT_)[m] (i.e., by offset correction of preperturbation positions). Note that these, together with COM positions, inertia, and masses, are the inputs needed for defining the rigid bodies in SimMechanics. Additionally, *C*_a_ defines the position of the ankle joint, and *C*_h_ defines the position of the hip joint, which both are rotational DOFs around the *y*-axis. Note that the motion of the spine (i.e., bending of the lumbar spine: L1 to L5) is also lumped into the “hip” DOF, a procedure which is only accepted as segment lengths which do not change “significantly.”

Finally, the angles, which are defined to be zero at the upright position, are calculated by the centers *z* components (*C*_*i*_^[*z*]^), according to

(B.2) $$\begin{array}{c} \Theta_{a}=\sin^{-1}\frac{\left(C_{h}^{\left[z\right]}-C_{a}^{\left[z\right]}\right)}{l_{Leg}},\\ \Theta_{h}=\gamma -\Theta_{a},with\;\gamma =\sin^{-1}\frac{\left(C_{s}^{\left[z\right]}-C_{h}^{\left[z\right]}\right)}{l_{HAT}}. \end{array}$$

#### B.3 MATLAB : Simulink : SimMechanics

In the SimMechanics model, the two rotational DOFs (Θ_A,H_) are represented by frictionless, unconstrained revolute joints. The third DOF of the model, on which the perturbation is acting (which is depicted by *x* in 1), is realized by a frictionless, unconstrained prismatic joint between the global CS position: (0 0 0.02)[m] (the initial position of the feet) and *C*_a_. The perturbation signal is created by using the vertical translation of the ankle, thus ignoring the transfer of the original perturbation through the feet. For the initialization of the model in SimMechanics, the influence of the noise, generated by the experimental setup and by the marker tracking, is reduced by using the mean of eight time steps, prior to the perturbation. The data is corrected for its global offset, such that the DIP is uprightly aligned above the global CS origin. The SimMechanics model then automatically adapts to the physique (see Figure 2*W*) of each participant (and trial).

### C. Solution of the Differential Equation for a Mechanical Eigenmovement in Alexandrov et al. [37]

The second order in time *t*, ordinary differential equations (2) in Alexandrov et al. [37]

(C.1) $$\begin{array}{c} -\lambda_{i}\cdot {\ddot{\xi }}_{i}\left(t\right)+\xi_{i}\left(t\right)=\eta_{i}\left(t\right), \end{array}$$

represent the mechanical dynamics of an open, nonramified kinematic chain exposed to gravity after linearization (see (2) in [37]) of the nonlinear, mechanical equations of motion ((1) in [37]) around the unstable equilibrium of all *N*-links being in upright position and after the expansion of the solution ((4) in [37]) of the linearized system of equations ((2) in [37]) into its eigenvectors *w*_*i*_ ((3, 10) in [37]). Here, *ξ*_*i*_(*t*) are the amplitudes of the (angular) eigenvectors *w*_*i*_ and *i* = 1,…, *N* is the index of the angular degrees of freedom of this *N*-link-inverted pendulum. The solution for (C.1) ((5) in [37]) is a superposition of the solution for (C.1) in its homogeneous case (right hand side is zero: *η*_*i*_(*t*) = 0) and the solution in its inhomogeneous case, that is, with the actual torque amplitudes *η*_*i*_(*t*) (see (5, 6) in [37]) acting along the eigenvectors *w*_*i*_

(C.2) $$\begin{array}{c} \xi_{i}\left(t\right)=c_{1,i}\cdot e^{t/\sqrt{\lambda_{i}}}+c_{2,i}\cdot e^{-t/\sqrt{\lambda_{i}}}+e^{-t/\sqrt{\lambda_{i}}}\cdot \int_{1}^{t}\frac{e^{x/\sqrt{\lambda_{i}}}\cdot \eta_{i}\left(x\right)}{2\sqrt{\lambda_{i}}}\;dx+e^{t/\sqrt{\lambda_{i}}}\cdot \int_{1}^{t}-\frac{e^{-x/\sqrt{\lambda_{i}}}\cdot \eta_{i}\left(x\right)}{2\sqrt{\lambda_{i}}}\;dx. \end{array}$$

The movement of the *N*-link-inverted pendulum nearby its unstable upright position is thus always a linear superposition of exponentially leaving or approaching this unstable equilibrium along specifically weighted combinations of joint angles (eigenvectors *w*_*i*_), with a characteristic time constant (eigenvalue *λ*_*i*_) for each eigenmovement (or: eigenmode) along the corresponding eigenvector. For “leaving” to occur, for example, when exposed to just gravity and with no initial velocities, the initial position must be adjacent to but not exactly at the ideal upright position. This is reflected by the time-inverted dynamics, which are also solutions, namely, when the kinetic energy in any mode is the same but the velocities inversely directed: an exponential movement is always just an *asymptotic convergence* to a final position. Therefore, if the initial kinetic energy is exactly tailored to overcome gravitational potential energy up to the unstable equilibrium, approaching the latter will occur asymptotically, taking infinitely long time.

### D. Phases in Balance Loss and Recovery

Alternate to Figure 4, the following text describes the whole lateral motion studied, that is, prior to the perturbation, the participant is standing in upright, hip-wide stance on the BaMPer system (a), as it was defined in the Method. At (a), for the participant unknown time, the perturbation is triggered by a shift of the platform to the left (or right). The resulting force is tilting the Leg segment (negative Θ_a_), leaving the upper body unaltered at first (positive Θ_h_); thus, the angles move “antiphase” (b). After a response time, in which the perturbation is acting unhindered (i.e., participant in free fall), the hip angle is returned to 0[°] (probably as a result from stiffening of the whole body, but especially the hip joint), which is the first visible active reaction of the participant to the perturbation (b to c), and here, a brief moment exists where both angles move in phase, primarily as a result of the active control and the still acting motion of the perturbation. The lateral translation of the lower segments (by the still moving platform) and the participants' reaction results in the CoP (whole-body COM projection to the BoS), moving to the edge of the BoS (and sometimes leaving it). The reaction (c), at first glance, seems to further enhance the loss of balance. However, on second glance, the legs are tilted back to upright stance. The hip angle, which is mostly moving antiphase to the ankle angle, aside from the brief moment mentioned above and for a longer period at the end of recovery, further shifts to the negative as a result of a “rapid hip movement” with the direction of perturbation. This second, distinctly stronger reaction straightens the legs completely and thus brings the ankle angle back to 0[°] (d). The hip angle which is still tilted is brought back to the upright initial position (e, f). Note that if the perturbation is to the opposite side, the angular trajectories are reversed as well.

#### Data Availability

All research material is available to the public for noncommercial use and can be provided if asked for. This contains experiment data (e.g., videos, marker positions in Excel) and simulation data (code).
